# Aminated Graphene as an Advantageous Filler for Polymer Composites with a Segregated Structure

**DOI:** 10.3390/nano16100584

**Published:** 2026-05-11

**Authors:** Kseniya A. Shiyanova, Mikhail K. Torkunov, Egor A. Inshakov, Sergei A. Ryzhkov, Maria Brzhezinskaya, Natalia G. Ryvkina, Igor A. Chmutin, Alexander S. Zabolotnov, Alexander A. Gulin, Oleg V. Uvarov, Demid A. Kirilenko, Sergey I. Pavlov, Maksim K. Rabchinskii

**Affiliations:** 1 Semenov Federal Research Center for Chemical Physics, Russian Academy of Sciences, 119911 Moscow, Russia; tmk19981207@yandex.ru (M.K.T.); ryv@mail.ru (N.G.R.); zabolotnov.ru@gmail.com (A.S.Z.); aleksandr.gulin@phystech.edu (A.A.G.); 2 Ioffe Institute, Politekhnicheskaya St. 26, 194021 Saint Petersburg, Russia; egor.inshakov@mail.ioffe.ru (E.A.I.); ryzhkov@mail.ioffe.ru (S.A.R.); zumsisai@gmail.com (D.A.K.); pavlov_sergey@mail.ioffe.ru (S.I.P.); 3 Nikolaev Institute of Inorganic Chemistry, Siberian Branch of the Russian Academy of Sciences, 630090 Novosibirsk, Russia; 4 Helmholtz-Zentrum Berlin für Materialien und Energie, Hahn-Meitner-Platz 1, 14109 Berlin, Germany; maria.brzhezinskaya@helmholtz-berlin.de; 5 Microsensorics Innovation and Engineering Center, MIREA-Russian Technological University, 119454 Moscow, Russia; tchmutin@mail.ru; 6 Prokhorov General Physics Institute, 119991 Moscow, Russia; uvarov@kapella.gpi.ru; 7 Advent, Ltd. 909, 28, Digital-ro 33-gil, Guro-gu, Seoul 08377, Republic of Korea

**Keywords:** copolymer of vinylidene fluoride and tetrafluoroethylene, polyvinyl chloride, AmG, electrically conductive composite, EMI protection

## Abstract

Conductive polymer composites with a segregated structure are a promising route to obtaining electrically active materials at low filler loadings. In this work, aminated graphene (AmG) was used as a functional conductive filler for the fabrication of composites with a segregated structure based on polyvinyl chloride (PVC) and poly(vinylidene fluoride-co-tetrafluoroethylene) (P(VDF-TFE)). AmG was comprehensively characterized by electron microscopy, core-level and near-edge spectroscopy, optical spectroscopy, and electrical measurements. The synthesized AmG contained 14.34 at.% nitrogen, with amines accounting for 81.44% of the nitrogen-related spectral intensity, corresponding to an amine concentration of 11.78 at.%. AmG also exhibited a restored π-conjugated network, intrinsic conductivity of 20–33 S/cm, and a crumpled-flake morphology favorable for interfacial contact with polymer particles. At a filler loading of only 1 wt.%, the segregated composites reached electrical conductivity up to 1.3–1.4 × 10^−4^ S/cm, exceeding those of the unfilled polymers by seven orders of magnitude. At 11 GHz, the AmG-filled P(VDF-TFE) composite showed 15.1 dB attenuation for a theoretical thickness of 30 mm, transmitting no more than 3% of the incident radiation. These results identify AmG as a functional conductive filler for segregated electrically conductive polymer composites and demonstrate that the combination of amine-containing surface chemistry, restored electrical conductivity, and crumpled morphology enables conductive interparticle network formation in PVC- and P(VDF-TFE)-based composites at only 1 wt.% filler loading.

## 1. Introduction

Electrically conductive polymer composites (ECPs) filled with graphene and its derivatives have attracted considerable attention due to their ability to combine the processability, mechanical flexibility, and low density of polymers with the high electrical conductivity, large specific surface area, and multifunctionality of carbon nanofillers. Owing to this combination of properties, ECPs show strong potential for a wide range of technical applications. They can be used as highly efficient filter membranes [1,2,3], electric heaters [4], and flexible conductive materials [5]. In electrical engineering, they are promising for energy generation devices [6] and for the fabrication of sensors and detectors [7,8,9]. Their outstanding performance in electromagnetic radiation shielding applications should also be noted [10,11].

The relevance of electromagnetic interference (EMI) shielding materials has increased due to the continuous growth of wireless communication systems, compact electronic devices, and high-density integrated electronics. Electromagnetic interference can cause signal distortion, malfunction, data loss, and degradation of electronic components. Conventional metal shields provide high shielding efficiency, but their high density, limited corrosion resistance, poor flexibility, and predominantly reflection-based shielding mechanism restrict their use in lightweight and flexible electronic systems. Polymer-based conductive composites are therefore actively studied as an alternative class of EMI-shielding materials because they combine low density, processability, corrosion resistance, and the possibility of structural design [12,13,14,15].

A key challenge in the development of conductive polymer composites is the statistically non-uniform distribution of conductive filler within the polymer matrix. This often leads to deterioration of important physical and mechanical properties and increases the cost of the final material, since relatively high filler loadings are typically required to reach the percolation threshold. Therefore, broader application of carbon-filled conductive polymer composites requires approaches that enable a small amount of filler to be efficiently organized into a continuous conductive network within the designed composite structure.

One of the most effective strategies is the preparation of composites with a segregated structure [16,17,18,19]. In such materials, the conductive filler is predominantly localized on the surface of polymer powder particles used as the particulate precursor of the matrix. During consolidation, these filler-coated powder particles are transformed into polymer domains separated by filler-rich interparticle regions. This results in a locally high concentration of the conductive phase, promotes the formation of conductive pathways, and significantly reduces the total filler content required to achieve electrical conductivity. Thus, the formation of efficient conductive architecture is more important than simply increasing the filler content. Although high filler loading can improve conductivity and electromagnetic interference (EMI) shielding, it also increases density, worsens processability, and may deteriorate mechanical properties.

Graphene is one of the most attractive carbon fillers for electrically conductive polymer composites because of its two-dimensional morphology, high aspect ratio, large specific surface area, extended sp^2^-conjugated structure, and high intrinsic electrical conductivity. These features allow graphene-based fillers to form percolated conductive pathways at relatively low filler contents, provided that aggregation and restacking are sufficiently suppressed. For EMI-shielding applications, however, the performance of graphene/polymer composites is determined not only by the intrinsic conductivity of graphene, but also by its spatial organization within the polymer matrix.

Recent studies on structurally organized graphene/polymer materials demonstrate that segregated, porous, layered, oriented, and hierarchically organized graphene-containing architectures can improve electromagnetic attenuation by combining conductive network formation with interfacial polarization, conduction-related dielectric loss, impedance matching, and internal reflection or scattering at heterogeneous interfaces [20,21,22]. This makes graphene-based fillers particularly relevant for lightweight conductive and EMI-shielding polymer composites, where efficient network formation at reduced filler loading is required.

At the same time, pristine graphene and reduced graphene oxide often tend to aggregate due to van der Waals interactions, while insufficient compatibility with polymer matrices can lead to non-uniform filler distribution and unstable conductive pathways. Chemical functionalization of graphene derivatives is therefore an important route to improve filler localization, interfacial adhesion, and the stability of conductive networks in polymer composites. In this regard, graphene oxide (GO) and its chemically modified derivatives are of particular interest, since GO provides a chemically tunable platform that can be functionalized in various ways [7], thereby imparting additional surface chemistry and application-specific properties. Importantly, chemical modification of GO, including amination or bromination, may enhance compatibility and interfacial interaction between the filler and the polymer matrix, facilitate more efficient filler distribution, and potentially reduce the required filler loading [23]. Another advantage of using functionalized graphene derivatives is that they may eliminate the need for a separate reduction step to convert GO into an electrically conductive form.

Among nanocarbon fillers, aminated graphene (AmG) has recently emerged as a particularly promising material because it combines the large specific surface area and electrical conductivity of graphene with chemically active amine groups, which improve filler dispersibility and interfacial interactions within polymer matrices [24,25]. In electrically conductive polymer composites, this combination is especially relevant because amine functionalities can enhance compatibility with the surrounding polymer phase, provide reactive surface sites for further covalent modification, grafting, and interface tailoring, and help suppress excessive agglomeration and restacking. As a result, AmG offers broader opportunities to control filler distribution and improve the efficiency of conductive network formation at reduced filler loading. In addition, the level of amine introduction can affect the electronic structure of graphene derivatives, including the work function and band structure [26]. This is relevant for EMI-shielding applications because these parameters influence charge-transfer and polarization behavior in AmG and in the composites derived from it.

The practical potential of AmG and other functionalized graphene derivatives has already been demonstrated in several composite systems. For example, EMI-shielding textile materials based on silver/polyaniline/AmG were developed in [10]. These modified fabrics exhibited EMI attenuation above 40 dB across ultra-high-frequency (UHF), L-, S-, C-, and X-bands, exceeding commercial requirements. In another study, amino-functionalized reduced graphene oxides and graphite-fluorinated polymers were introduced into polyurethane to enhance its triboelectric characteristics, resulting in an approximately 2.2-fold increase in the surface positive potential of the polymer matrix (from 201 V to 480 V) [27]. Functionalized graphene derivatives have also proven effective in protective composite coatings: GO functionalized with 4-(trifluoromethyl)benzohydrazide improved the barrier properties of phenolfurfural polymer-based coatings, lowered the curing temperature, and provided durable corrosion protection under prolonged exposure to saline media [28]. These examples demonstrate that appropriate graphene functionalization can strongly affect not only the electrical performance of composites but also their interfacial, triboelectric, and barrier properties.

In this study, we investigated AmG as a functional filler for electrically conductive polymer composites with a segregated structure. Particular attention was paid to the relationship between the intrinsic properties of AmG, including its surface chemistry, restored π-conjugated graphene network, electrical conductivity, and crumpled high-surface-area morphology, and its behavior in composite systems based on polymer matrices with different particle morphologies. To this end, composites were prepared using two polymers, polyvinyl chloride (PVC), characterized predominantly by rounded particles, and poly(vinylidene fluoride-co-tetrafluoroethylene) (P(VDF-TFE)), possessing a highly developed tangled fibrous structure. Owing to their structures, where AmG is localized at polymer–particle interfaces and forms a segregated conductive network after hot pressing, the fabricated and studied composite materials are defined as segregated AmG-filled ECPs.

The aim of this work was to determine how the physicochemical characteristics of AmG affect its localization at polymer particle interfaces, the formation of segregated conductive pathways during AmG-filled ECP consolidation, and the resulting electrical and EMI-shielding performance of the materials. The main scientific contribution of this work is the demonstration of AmG as a functional filler for segregated electrically conductive polymer composites, where amine-containing surface chemistry, restored electrical conductivity, and crumpled morphology jointly enable AmG localization at polymer–particle interfaces and conductive network formation at only 1 wt.% filler loading.

## 2. Materials and Methods

### 2.1. AmG and rGO Synthesis

AmG was prepared from GO by reductive amination using a modified Leuckart reaction, as described previously [29,30]. Briefly, 100 mL of a 1 wt.% aqueous GO suspension was transferred into a Teflon-lined reaction vessel and combined with 75 mL of formamide (Merck KGaA, Darmstadt, Germany). The resulting mixture was heated to 165 °C and maintained under continuous magnetic stirring at 250 rpm for 48 h. After completion of the reaction, the suspension was allowed to cool to room temperature and then filtered through a glass filter with a pore size of 40 μm to isolate the AmG powder. The collected product was thoroughly washed with deionized water (three times) and isopropyl alcohol (three times).

The GO used in this work was synthesized by a modified Hummers method, yielding an aqueous dispersion with a concentration of 10 mg/mL, as reported in Ref. [19].

### 2.2. AmG-Filled ECP Fabrication

To fabricate the AmG-filled ECPs, an aqueous dispersion of AmG was first prepared. To ensure a uniform distribution of AmG in the solvent, the suspension was treated with an immersion ultrasonic processor at 600 W for 40 min, with 5 min pauses after every 10 min of sonication. Cooling was provided using an ice bath. The polymer was introduced during the final 10 min of ultrasonic treatment. Two polymers were used as the matrix materials: poly(vinylidene fluoride-co-tetrafluoroethylene), P(VDF-TFE) (trade name F-42V, GOST 25428-82, HaloPolymer OJSC, Moscow, Russia), and polyvinyl chloride (PVC) (trade name PVCS-7059M, GOST 14332-78, Ruskhim LLC, Ufa, Russia). For both polymer matrices, the filler content was fixed at 1 wt.%. After sonication, the resulting dispersion was filtered through a funnel equipped with a Schott filter (pore size 16). The wet polymer powder coated with AmG was then dried in air in a drying oven at 80 °C for 24 h. The fabricated samples are summarized in Table 1.

### 2.3. GO and AmG Characterization

AmG films on Si and quartz substrates, TEM grids, and multi-electrode chips for spectroscopic, microscopic, and electrical characterization were prepared by spray-coating from an isopropanol suspension of AmG with a concentration of 0.1 mg/mL, following the procedure described in Ref. [30]. GO films were deposited in the same manner using an aqueous GO suspension with a concentration of 0.05 mg/mL.

#### 2.3.1. XPS and XAS Studies

The chemical composition of the initial GO and AmG samples was analyzed by X-ray photoelectron spectroscopy (XPS) and X-ray absorption spectroscopy (XAS) at the ultra-high-vacuum experimental station of the Russian–German beamline at the BESSY-II electron storage ring, Helmholtz-Zentrum Berlin (HZB) [31]. Prior to the measurements, all samples were evacuated at a pressure of ~10^−9^ Torr for 24 h to remove adsorbed species. No thermal treatment was applied in order to preserve the original chemical state of the materials. The XPS and XAS spectra were recorded from four equidistant areas separated by approximately 500 µm, each with a size of about 200 × 100 µm. For both GO and AmG, the variation between spectra collected from different positions did not exceed 4%, indicating good chemical uniformity of the samples. The averaged spectra were then used for quantitative analysis.

The survey, C 1s, and N 1s XPS spectra were acquired at an excitation energy of 850 eV, with a pass energy of 20 eV and an energy step of 0.5 eV for the survey spectra and 0.05 eV for the C 1s and N 1s core-level spectra. Elemental atomic concentrations were determined from the survey spectra using the relative sensitivity factors C = 1, O = 2.93, and N = 1.80. Further deconvolution of the C 1s and N 1s spectra was performed using CasaXPS software (Version 2.3.16Dev52, Casa Software Ltd., Teignmouth, UK). All spectra were fitted using a Shirley background, while different line-shape models were applied for different spectral regions. The C 1s spectra were fitted using one asymmetric Doniach–Šunjić function (DS; 0.09–0.15; 90–250; GL90) and five symmetric Gaussian–Lorentzian functions with a 70:30 ratio (GL(30)). The N 1s spectra were deconvoluted using four symmetric Gaussian–Lorentzian functions with the same 70:30 ratio (GL(30)).

The C K-edge and N K-edge X-ray absorption spectra were recorded in the total electron yield mode, corresponding to a probing depth of approximately 10 nm, at the magic angle (α = 54.7°), which provides comparable contributions from the π- and σ-related states. The acquired XAS spectra were normalized and smoothed according to the procedure described elsewhere [32].

#### 2.3.2. Raman Spectroscopy

The morphology and structural features of GO and AmG were investigated by Raman spectroscopy, scanning electron microscopy (SEM), and transmission electron microscopy (TEM), complemented by electron diffraction (ED). Raman spectra were recorded in the backscattering geometry using a Horiba LabRAM HREvo UV-Vis-NIR-Open spectrometer (Horiba Jobin-Yvon, Lille, France). An Nd:YAG laser (Torus, Laser Quantum, Inc., Stockport, UK) operating at λ = 532 nm (E = 2.33 eV) was used as the excitation source. The laser power was limited to 0.4 mW to avoid sample damage. The beam was focused to a spot smaller than 1 μm using an Olympus MPLN 100× objective (NA = 0.9). The acquired Raman spectra were deconvoluted using four to six components fitted with either Lorentzian functions (D, G, and D′ bands) or Gaussian functions (D3, 1D chains, and C=O/C=C-related bands).

#### 2.3.3. SEM&TEM Imaging

SEM images of Si wafers covered with arrays of individual GO and AmG flakes were acquired using a JSM-7001F microscope (Jeol, Tokyo, Japan). TEM images and ED patterns were obtained from individual GO and AmG platelets aerosol-deposited onto TEM Cu grids (400 mesh) using a JEM-2100F microscope (Jeol, Ltd., Tokyo, Japan).

#### 2.3.4. Electrical Conductivity Measurements

To evaluate the electronic properties of GO and AmG, electrical resistivity measurements using a four-point probe configuration were combined with ultraviolet–visible (UV–Vis) spectroscopy. For the electrical measurements, the AmG film was deposited onto quartz substrates equipped with four Au electrodes 80 nm in thickness and separated by 1 mm gaps. The current–voltage (I–V) characteristics were recorded in the voltage range from −2 to +2 V at room temperature using a Keithley 6487 picoammeter/voltage source (Keithley Instruments, Solon, OH, USA).

#### 2.3.5. UV-Vis Studies

UV–Vis measurements were carried out for GO and AmG films deposited on quartz substrates using a Shimadzu UV-2450 spectrometer (Shimadzu, Kyoto, Japan) without an integrating sphere [33]. The optical density spectra were recorded in the wavelength range of 190–800 nm with a step size of 0.5 nm.

### 2.4. Composite Characterization

#### 2.4.1. SEM Imaging

SEM was employed to examine the surface topography of powders modified with AmG. The measurements were performed using a Prisma E microscope (Thermo Fisher Scientific, Brno, Czech Republic). Prior to imaging, the powder particles were fixed onto carbon adhesive tape and coated with a 10 nm Au layer using a Q150RES sputter coater (Quorum Technologies, Laughton, UK) to minimize surface charging. The observations were carried out under high-vacuum conditions at an accelerating voltage of 2–5 kV. During the measurements, the sample was mounted on an L-shaped holder with its surface oriented perpendicular to the optical axis of the electron beam.

#### 2.4.2. Ultramicrotome Probing

A Leica EM UC6 ultramicrotome (Leica Microsystems, Wetzlar, Germany) was used to prepare thin sections of the fabricated samples. Sectioning was carried out at room temperature to obtain slices with a thickness of 1000 nm. The prepared sections were subsequently examined using a Zeiss Axiotech 100 HD optical microscope (Zeiss, Oberkochen, Germany).

#### 2.4.3. Impedance Spectroscopy Studies

The electrical conductivity of the AmG-filled ECPs was studied using an LCR-78105G dielectric spectrometer (Good Will Instrument Co., New Taipei City, Taiwan). The AmG-filled ECPs based on P(VDF-TFE) and PVC were hot-pressed under the following conditions: 200 °C and 140 kg/cm^2^ for P(VDF-TFE) and 120 °C and 300 kg/cm^2^ for PVC. As a result, disk-shaped samples with a diameter of 12 mm and a thickness of 1 mm were obtained. To ensure reliable electrical contact, both surfaces of the samples were coated with a silver-based conductive adhesive (MCN-DJ002, Mechanic Company, Shenzhen, China) with a conductivity of 10^4^ S/cm. Impedance spectroscopy measurements were performed over the frequency range from 20 Hz to 5 MHz.

#### 2.4.4. Microwave Permittivity Measurements

The dielectric properties of the materials in the microwave range at 11 GHz were investigated using a resonator-based technique. The experimental setup comprised a rectangular resonator coupled to a panoramic standing wave ratio (SWR) meter (JSC NPK MERA, Krasnodar, Russia). Rectangular parallelepiped samples with dimensions of 5 × 1 × 1 mm were used to ensure proper positioning within the resonator. During the measurements, the real (ε′) and imaginary (ε″) components of the complex permittivity (ε*), as well as the absorption (A), reflection (R), and transmission (T) coefficients, were determined. The resonator-based setup used in this work operates at a fixed frequency of 11 GHz. Therefore, the measurements provide single-frequency microwave characteristics and do not represent continuous broadband data over the full X-band.

#### 2.4.5. Mechanical Property Characterization

The deformation and strength properties of the AmG-filled ECPs were determined in the uniaxial tensile mode of specimens on an INSTRON 3365 testing machine (Instron Limited, Norwood, MA, USA) at room temperature (~21 ± 1 °C) (type 5, equivalent length 50 mm) in accordance with GOST 11262 and GOST 9550. The strain rate of the specimens under tension was 0.02 min^−1^ (the speed of expansion of the clamps of the testing machine was 1 mm × min^−1^ to determine the modulus at the initial 1st stage) and 1.0 min^−1^ (the speed of expansion of the clamps of the testing machine was 50 mm min^−1^ at stage 2). Specimens in the form of double-sided blades were cut out using a special cutting knife. Mechanical properties were determined for five specimens of each P(VDF-TFE)-based material and are reported as mean ± standard deviation.

## 3. Results

### 3.1. AmG Chemistry

**Figure 1:**
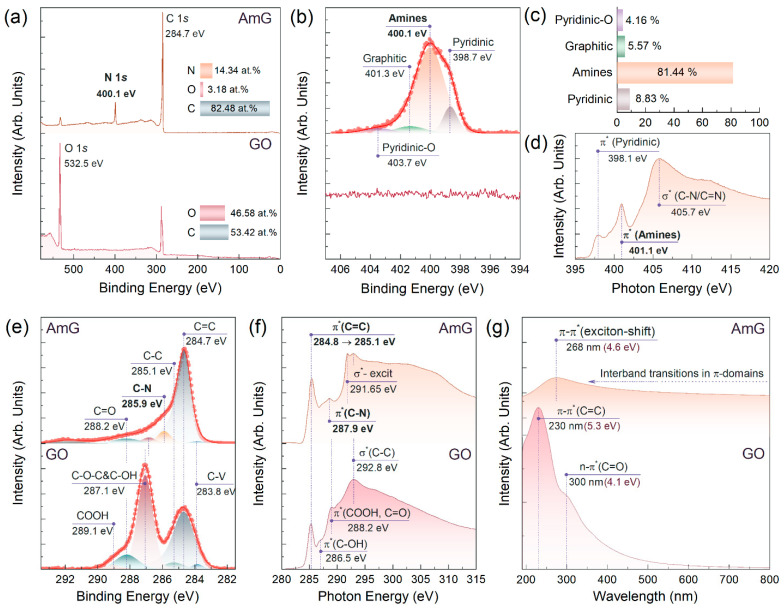
Examination of the chemistry and electronic structure of the synthesized AmG. (**a**) Survey and (**b**) high-resolution N 1s XPS spectra of GO and AmG; (**c**) bar plot demonstrating the relative concentration of the nitrogen-containing groups in AmG; (**d**) N K-edge XAS spectrum of AmG; (**e**) C 1s XPS, (**f**) C K-edge XAS, and (**g**) UV-Vis spectra of the GO and AmG.

Figure 1a exhibits the survey XPS spectra of the initial GO and the synthesized AmG. The pronounced decrease in the oxygen content, accompanied by the appearance of nitrogen-related spectral features, clearly indicates successful amination, as evidenced by the evolution of the O 1s and N 1s signals. Notably, the oxygen concentration decreases from 46.58 at.% in GO to 3.18 at.% in AmG, while the nitrogen content reaches 14.34 at.%. Apart from the C 1s peak, no additional signals are detected, indicating the high chemical purity of the synthesized AmG.

To clarify the nature of the nitrogen-containing species in AmG, the N 1s spectrum was further analyzed by peak deconvolution, as displayed in Figure 1b. Four components centered at 398.7, 400.1, 401.3, and 403.7 eV were identified and assigned to pyridinic nitrogen, amine groups, graphitic nitrogen, and pyridinic-N-oxide species, respectively [34,35]. The amine-related component clearly dominates the N 1s envelope, accounting for 81.44% of the total spectral intensity, whereas the contributions of pyridinic and graphitic nitrogen are substantially lower, amounting to 8.83% and 5.57%, respectively (Figure 1c). Based on these data, the atomic concentration of amine groups in the synthesized AmG was estimated at 11.68 at.%, which is among the highest values reported to date [35,36,37].

The XPS results are in good agreement with the acquired XAS data. As shown in Figure 1d, the N K-edge XAS spectrum of AmG is dominated by the π* resonance of amine groups centered at 401.1 eV [26,35]. In contrast, the π* resonance associated with pyridinic nitrogen at 398.1 eV is much less intense, while the π* resonance of pyrrolic species, typically observed at 399.2 eV [38], is not detected. This supports the assignment of the 400.1 eV component in the N 1s spectrum to amines rather than pyrroles, since these two nitrogen-containing species exhibit close binding energies [35,38,39].

Elimination of the oxygen-containing groups with the restoration of the π-conjugated graphene network upon conversion of GO into AmG is signified by further examination of the C 1s XPS and C K-edge XAS spectra. Drastic diminishing of the spectral features related to the basal-plane hydroxyls and epoxides, ketones, and carboxyls positioned at 287.1 eV, 288.2 eV, and 289.1 eV [40,41,42] is indicated upon moving from GO to AmG in the C 1s spectra displayed in Figure 1e. Conversely, the asymmetric C=C peak related to carbon atoms, comprising a π-conjugated graphene network, becomes the dominating one in the case of AmG, with negligible contribution from the C-V peak at 283.8 eV and C-C peak at 285.1 eV, related to carbon atoms at vacancy defects and aliphatic carbon, respectively [40,43].

### 3.2. AmG Electronic Structure and Conductivity

These observations are consistent with the evolution of the C K-edge XAS spectra after reductive amination of GO. As presented in Figure 1f, the π*(C=C) resonance shifts toward higher photon energies, from 284.8 eV for GO to 285.1 eV for AmG, and its intensity increases relative to that of the σ*(C=C) resonance at 292.8 eV [42]. In addition, a clearly resolved σ*-exciton peak appears at 291.65 eV [32,35]. Taken together, these spectral changes indicate that AmG contains extended regions of a restored π-conjugated graphene network while retaining a high concentration of introduced amine groups.

The high degree of restoration of the π-conjugated graphene network in AmG is also reflected in its optical and electrical properties. As shown in Figure 1g, AmG exhibits strong optical absorption throughout the visible and near-ultraviolet spectral range, with only weak wavelength dependence, which is consistent with substantial band-gap suppression upon reductive amination. The only pronounced spectral feature is an absorption peak centered at 268 nm, which is attributed to excitonic effects associated with band-to-band transitions [44]. This behavior differs markedly from that of GO, which shows almost no optical absorption at wavelengths above 400 nm due to the presence of a band gap of ~2.5–3.1 eV. In the GO spectrum, only two prominent absorption bands can be distinguished, located at 230 and 300 nm and commonly assigned to interband π–π* transitions in localized C=C bonds and n–π* transitions of C=O groups, respectively [45].

The restoration of the conjugated network is further confirmed by the electrical measurements. According to the four-point probe data, the resistivity decreases drastically from 10^10^–10^11^ Ω/m for GO to 0.3–0.5 × 10^−3^ Ω/m for the AmG film, corresponding to a conductivity of 2000–3300 S/m. These results indicate that the applied reductive amination not only introduces a high concentration of amine groups, but also effectively converts GO into an electrically conductive graphene derivative.

### 3.3. AmG Morphology

**Figure 2:**
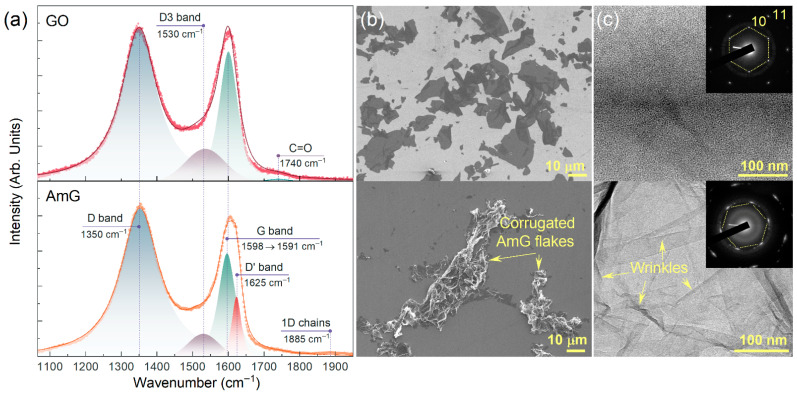
Probing the morphology of AmG. (**a**) Raman spectra, (**b**) SEM images, and (**c**) TEM images of GO (upper line) and AmG (bottom line). Insets—ED patterns acquired from the areas presented in the TEM images.

Characterization of the AmG chemistry and electronic structure was further complemented by morphological analysis in comparison with the initial GO. Figure 2a displays the Raman spectra of the studied materials, which include the *D*, *D3*, *G*, and *D′* bands centered at 1350 cm^−1^, 1530 cm^−1^, 1591–1598 cm^−1^, and 1625 cm^−1^, respectively. The G band originates from the doubly degenerate *E*_2g_ vibrational mode of sp^2^-bonded carbon atoms, whereas the *D* band is associated with a defect-activated double-resonance scattering process involving a transverse optical (TO) phonon with a non-zero wave vector and lattice defects in graphene [46]. The specific origin of the *D3* band has not yet been unambiguously established in the literature; however, it is commonly associated with disordered or amorphous carbon regions [46,47]. The *D* and *D′* bands are both defect-related, while the *D3* contribution may also reflect the presence of local amorphized carbon domains and/or adventitious carbon contamination [46,47].

In addition to these bands, the GO spectrum exhibits a C=O-related feature centered at 1740 cm^−1^, which is most likely associated with vibrational modes of carbonyl groups in ketones and ethers [48]. This band disappears after amination, indicating substantial removal or transformation of oxygen-containing functionalities. In turn, the AmG spectrum shows the emergence of the 1D-chain band at 1885 cm^−1^ together with the *D′* band at 1625 cm^−1^. The former is typically attributed to linear aliphatic carbon species, including residual alcohol-derived fragments or amorphized graphene regions [49]. The *D′* band corresponds to another defect-activated double-resonance process involving the same phonon branch as the *D* band, but with a smaller wave vector [46].

As can be seen, the I_D_/I_G_ ratio and the full width at half maximum of the *D* band remain nearly unchanged after GO reductive amination, whereas the intensity of the *D′* band, which is inversely related to the content of sp^3^-hybridized carbon, increases. This indicates that most oxygen-containing groups are removed during amination, while also suggesting the formation of vacancy-type defects and distortions in the graphene lattice induced by amine incorporation and, in particular, by folding of the AmG sheets [49]. This conclusion is further supported by the SEM images shown in Figure 2b. The initially lamellar and relatively flat GO flakes become strongly crumpled after amination, exhibiting numerous folds with heights reaching several micrometers, regardless of the deposition method or solvent used. This behavior arises from the removal of basal-plane oxygen groups, which alters the balance of forces governing layer planarity, together with lattice distortions introduced by amine functionalization [50,51]. As a result, folding of the graphene sheets is promoted as a mechanism for strain relaxation.

Thus, the restoration of the conjugated network and the presence of defect-related Raman features should be considered complementary rather than contradictory. XPS, XAS, UV-Vis spectroscopy, and electrical measurements indicate removal of oxygen-containing groups and recovery of extended sp^2^-conjugated regions relative to GO. At the same time, Raman spectroscopy remains sensitive to local disorder; therefore, the nearly unchanged ID/IG ratio and increased D′ contribution indicate residual defects, amine-related lattice distortions, and folding-induced structural disorder. AmG should therefore be regarded as a conductive but defect-containing graphene derivative rather than defect-free graphene.

The TEM image shown in Figure 2c further confirms the corrugation of the graphene layers after amination. In particular, the AmG flakes exhibit a large number of folds that are not observed in the initial GO samples. The evolution of the electron diffraction (ED) patterns provides additional support for this conclusion. For both graphene derivatives, the ED patterns contain a single set of six (10) and (11) diffraction spots, indicating their predominantly monolayer character and the preservation of long-range ordering within the graphene lattice. At the same time, the diffraction spots become noticeably blurred in the case of AmG compared with GO, which can be attributed to variations in the angle between the electron beam and the local graphene surface caused by folding of the sheets [51]. Thus, although AmG demonstrates a high degree of restoration of the π-conjugated graphene network, it also possesses a complex crumpled morphology characterized by numerous folds and, consequently, a high specific surface area.

The absence of flat sheet-like flakes in the SEM and TEM images is attributed to the strongly crumpled morphology of AmG after reductive amination. The graphene layers are folded and corrugated and therefore appear as wrinkled, locally overlapped structures rather than extended flat platelets. The ED patterns nevertheless confirm preservation of graphene lattice ordering, while the blurred diffraction spots are consistent with local variations in sheet orientation caused by folding.

As a net result, the performed analysis shows that AmG combines three key features important for its use as a conductive polymer filler: a high concentration of surface amine groups, a substantially restored π-conjugated graphene network, and a highly developed crumpled morphology with numerous folds. The first factor provides chemically active sites and improved interfacial compatibility with the polymer matrix, whereas the latter two contribute to high electrical conductivity and potentially efficient formation of conductive networks.

### 3.4. Morphology of the AmG-Filled ECPs

**Figure 3:**
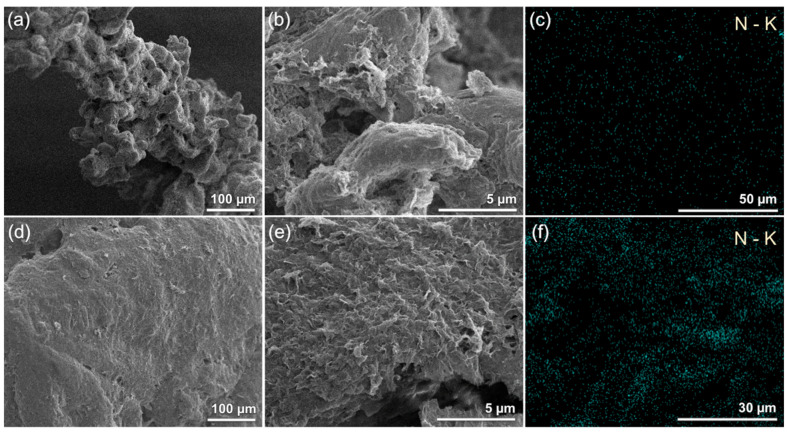
Representative SEM images and EDS nitrogen maps of polymer powder particles coated with AmG: (**a**–**c**) P(VDF-TFE)/AmG and (**d**–**f**) PVC/AmG.

To further assess the efficiency of AmG as a filler for segregated electrically conductive polymer AmG-filled ECPs, the surface morphology of the modified polymer powders was examined by SEM, while the presence and spatial distribution of AmG on the particle surface were verified by EDS mapping. Representative SEM images of the P(VDF-TFE)/AmG and PVC/AmG AmG-filled ECPs are shown in Figure 3. In both cases, AmG forms a dense surface coating on the polymer particles, indicating that the applied deposition procedure ensures efficient immobilization of the graphene derivative on matrices with markedly different particle morphologies. In the case of P(VDF-TFE) (Figure 3a,b), despite the complex and highly developed surface geometry of the polymer particles, the AmG coating remains continuous over large areas and follows the underlying relief well. For PVC (Figure 3d,e), which is characterized by more compact particle morphology, the filler also forms a pronounced surface layer, providing a nearly uniform coverage of the particle surface.

At higher magnification, the deposited AmG can be distinguished as a developed wrinkled and folded surface phase rather than as isolated compact inclusions. Such morphology is consistent with the intrinsic crumpled structure of AmG established above and is important from the standpoint of AmG-filled ECP formation. First, it increases the effective interfacial contact area between the filler and the polymer matrix. Second, it facilitates the formation of an interconnected conductive framework localized at the particle surface. Third, the absence of large compact agglomerates in the selected representative images indicates that AmG remains sufficiently well dispersed during processing and does not lose its surface-developed morphology upon deposition onto the polymer particles.

The SEM observations are further supported by the EDS elemental mapping displayed in Figure 3c,f. Since the unfilled polymer matrices do not contain nitrogen, the detected N signal can be directly attributed to AmG. For both polymer systems, nitrogen is detected over the particle surface, confirming the presence of AmG in the outer interfacial layer of the powder particles. Particularly, 10.0 ± 1.5 at.% of nitrogen is indicated for the P(VDF-TFE)/AmG AmG-filled ECPs and 14.6 ± 2.0 at.% for the PVC/AmG one.

The observed distribution of nitrogen indicates that the graphene derivative is not present as isolated random fragments but is associated with the polymer surface over extended areas. This result is especially important for segregated conductive composites, because it confirms the localization of the filler exactly where it is needed to form conductive pathways during subsequent consolidation of the powder. It should be emphasized that the target of the selected processing route is not homogeneous dispersion of AmG within the polymer bulk but rather uniform surface localization of a low amount of AmG on pre-existing polymer powder particles. The interaction between AmG and the polymer matrices is expected to be mainly interfacial and physical/polar in nature, involving the amine-containing surface of AmG and polar groups or dipoles of the polymer matrices, including C–Cl groups in PVC and C–F/C–H dipoles in P(VDF-TFE). In turn, no covalent polymer–filler bonding is assumed since such an interaction requires additional chemical treatment for the process to occur, hindering the simplicity of the composite processing method proposed.

**Figure 4:**
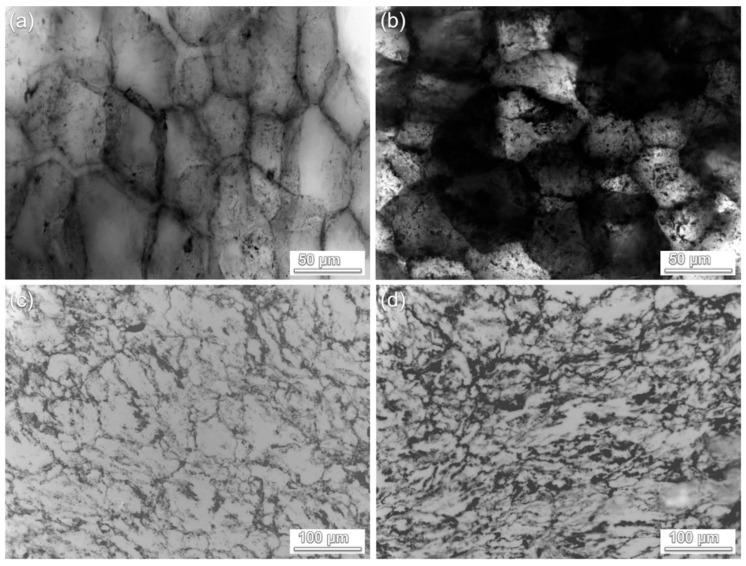
Optical images of ultramicrotomed cross-sections of pressed AmG-filled ECPs: (**a**,**b**) PVC/AmG and (**c**,**d**) P(VDF-TFE)/AmG.

The emphasized non-random and spatially extended localization of AmG on the polymer particle surface is further confirmed by the analysis of ultramicrotomed cross-sections of the formed AmG-filled ECPs. After pressing the AmG-filled ECP powders into disks, thin sections with a thickness of 1000 nm were prepared and examined by optical microscopy (Figure 4). In the PVC-based AmG-filled ECP, the cross-sectional images presented in Figure 4a,b clearly reveal a segregated structure, in which individual polymer particles are surrounded by filler-rich interfacial regions. This supports the conclusion from the SEM and EDS results that AmG remains associated with the particle surface over extended areas and, after consolidation, forms an interparticle conductive framework, as now demonstrated on a larger scale.

Moving to the P(VDF-TFE)-based AmG-filled ECP (Figure 4c,d), unambiguous visualization of the segregated structure is more challenging because the initial polymer particles possess a highly developed fibrous morphology. Nevertheless, the optical images of the acquired sections indicate that the conductive phase is distributed non-uniformly but remains localized within the interfacial regions of the composite rather than being homogeneously dissolved in the polymer bulk. Such behavior is consistent with the surface localization of AmG established above for the AmG-filled ECP powders and reflects the effect of the complex morphology of the fluoropolymer particles on the geometry and thickness of the filler-rich regions.

Thus, the formation of the segregated structure is primarily determined by the selected processing route. AmG is not introduced into a polymer melt as randomly dispersed isolated filler particles; instead, it is first deposited onto the surface of pre-existing polymer powder particles. During subsequent hot pressing, these AmG-coated particle surfaces are transformed into filler-rich interparticle regions, producing a segregated conductive network. The crumpled high-surface-area morphology of AmG and the presence of chemically active amine groups facilitate stable localization of the filler at the polymer–particle interface and support the formation of continuous conductive pathways at low filler loading.

### 3.5. AmG-Filled ECP Electrophysical Properties

**Figure 5:**
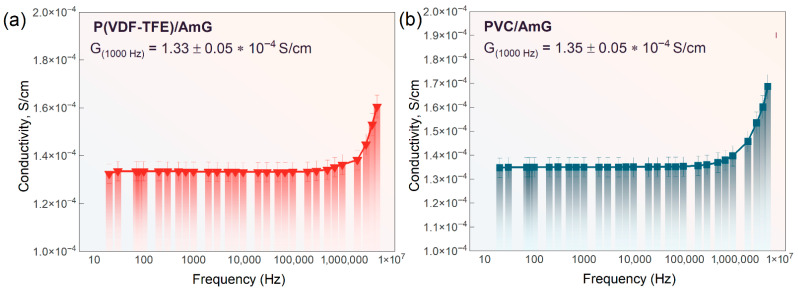
Frequency dependences of electrical conductivity for AmG-filled ECPs based on (**a**) P(VDF-TFE) and (**b**) PVC filled with AmG.

The indicated localization of AmG in extended interparticle regions creates favorable conditions for the formation of a continuous conductive network during hot pressing, even at low filler loading. This is reflected in the acquired frequency dependences of conductivity for the P(VDF-TFE)- and PVC-based composites, as shown in Figure 5. At 1000 Hz, the conductivity of the AmG-filled ECPs was in the range of (1.3–1.4) × 10^−4^ S/cm, with a standard deviation not exceeding 4% for three independently prepared specimens. This value is approximately seven orders of magnitude higher than that of the corresponding unfilled matrices.

Importantly, despite the pronounced differences in particle morphology between PVC and P(VDF-TFE), both systems demonstrate the same maximum conductivity level. This suggests that, within the studied compositions, the decisive factor governing charge transport is not the intrinsic shape of the polymer particles itself, but the ability of AmG to remain concentrated in the interfacial regions and to form a connected percolation network upon pressing and AmG-filled ECP formation. Therefore, AmG can be regarded as an efficient filler for the fabrication of electrically conductive polymer composites with a segregated structure, particularly in applications where moderate conductivity is sufficient, including antistatic materials.

### 3.6. AmG-Filled ECP EMI Properties

High electroconductivity of the fabricated AmG-filled ECPs suggests their possible advantageous use for EMI shielding applications. To evaluate this possibility, the measured real and imaginary parts of the complex permittivity were used to calculate the reflection, transmission, and absorption coefficients of microwave radiation at 11 GHz according to Equations (1)–(7), as detailed below [52]:

(1) $$R=\frac{{\left(n-1\right)}^{2}+k^{2}}{{\left(n+1\right)}^{1}+k^{2}}$$

where *R* is the reflection coefficient and *n* and *k* are calculation constants.

(2) $$T={\left(1-R\right)}^{2}\times e^{\left(-\alpha *h\right)}$$

where *T* is the transmission coefficient, *R* is the reflection coefficient, *α* is the calculation constant, and *h* is the thickness of the sample under study.

The absorption coefficient was calculated from the known relationship that the coefficients of reflection, transmission and absorption add up to unity (Equation (3)).

(3) A=1−T−R

The calculated parameter *n* was calculated from Equation (4). The calculated parameter *k* was calculated from Equation (5). The calculated parameter *α* was calculated using Equation (6).

(4) n=ε′2×1+(ε″ε′)2+1

(5) k=ε″(2·n)

(6) $$\alpha =\frac{k4\pi }{\lambda }$$

**Figure 6:**
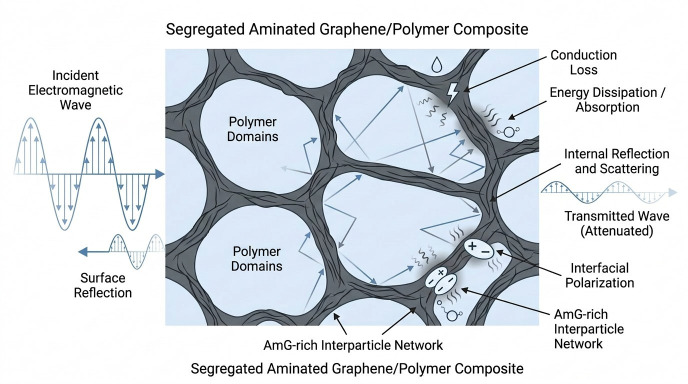
Schematic illustration of the EMI shielding mechanism in segregated AmG/polymer composites.

For electromagnetic waves in a vacuum, the speed of propagation is equal to the speed of light (299,792,458 m/s) and the wavelength $\lambda =\frac{299792458}{f}$. If *f* is expressed in hertz, then *λ* will be expressed in meters. The frequency of microwave radiation was chosen as 11 GHz. Accordingly, the calculated reflection, transmission, and absorption coefficients should be interpreted as parameters obtained for a single-frequency resonator measurement at 11 GHz, rather than as broadband shielding characteristics in the 8–12 GHz range. Figure 6 displays a schematic illustration of the EMI shielding mechanisms.

After obtaining the absorption, resistance and transmission values, they were converted to dB (Table 2) using Equation (7).

(7) $$T\;\left(dB\right)=10\times LG(T)$$

Among the studied materials, the P(VDF-TFE)-AmG AmG-filled ECP demonstrates the most pronounced microwave attenuation. At a theoretical thickness of 30 mm, this sample is characterized by ε′ = 3.18 and ε″ = 0.78, with calculated reflection, transmission, and absorption values of −11.9, −15.1, and −0.4 dB, respectively. In comparison, the PVC-AmG AmG-filled ECP shows lower dielectric losses and weaker attenuation, with ε′ = 2.36, ε″ = 0.22, and a transmission value of −6.9 dB. The unfilled P(VDF-TFE) matrix also exhibits noticeably inferior shielding performance relative to the AmG-filled ECPs, despite a comparable real permittivity, indicating that the introduction of AmG primarily enhances the dissipative component of the electromagnetic response.

These results are consistent with the structural model established above. In the present composites, AmG is localized in spatially extended interparticle regions and forms a connected conductive framework after consolidation. Such segregated architecture produces numerous polymer/AmG/polymer interfaces, which can promote partial internal reflection and scattering of the electromagnetic wave at filler-rich interparticle boundaries. However, multiple internal reflections should not be considered as the only or dominant shielding mechanism. The microwave response of the composites is more appropriately described as a combined result of partial surface reflection, internal reflection/scattering at heterogeneous interfaces, interfacial polarization, and conduction-related dielectric loss within the interconnected AmG network. Accordingly, the filler-induced conductive framework suppresses wave transmission, and the observed EMI performance can be regarded as a direct consequence of the segregated architecture formed by AmG in the polymer matrix.

An important result is that the P(VDF-TFE)-AmG composite functions predominantly as an absorbing rather than purely reflecting screen. This conclusion follows from the conversion of the logarithmic parameters in Table 2 to linear power fractions. For this composite, R = −11.9 dB and T = −15.1 dB correspond to approximately 0.065 and 0.031 of the incident power, respectively. Therefore, the absorption fraction estimated from A = 1 − R − T is approximately 0.904, i.e., about 90% of the incident electromagnetic power is dissipated within the material. The absorbance value close to 0 dB in Table 2 therefore does not indicate weak absorption; it reflects the logarithmic representation of a high linear absorption fraction. Such behavior is consistent with conductive loss and interfacial polarization within the AmG-rich segregated network and is advantageous for EMI materials because it reduces transmitted power while limiting secondary reflection. A comparison of the achieved results with relevant literature data is displayed in Table 3.

It should also be noted that unfilled PVC could not be evaluated under the same conditions because the material crumbled during sample preparation, and it was therefore impossible to obtain specimens of the required dimensions. Nevertheless, the available data clearly demonstrate that AmG is a viable filler not only for increasing the electrical conductivity of polymer composites but also for imparting microwave attenuation capability, making such materials attractive for EMI shielding applications.

### 3.7. AmG-Filled ECP Mechanical Properties

To complete the comprehensive characterization of the developed AmG-filled ECPs and consider their prospective use as structural elements for EMI shielding, their mechanical behavior was also evaluated. Tensile tests were performed for the P(VDF-TFE)-based materials, whereas PVC-based AmG-filled ECPs were not tested because the unplasticized PVC matrix was too brittle to prepare specimens of the required geometry. Mechanical properties were therefore determined for unfilled P(VDF-TFE) and for the P(VDF-TFE)-based AmG-filled ECP containing 1 wt.% AmG (Table 4).

The introduction of AmG leads to a noticeable change in the mechanical response of the fluoropolymer matrix. In particular, the elastic modulus increases from 613 ± 24 to 674 ± 37 MPa, whereas the tensile strength decreases from 52 ± 6 to 27 ± 2 MPa, and the elongation at break decreases from 346 ± 22 to 180 ± 14%. Thus, incorporation of AmG makes the composite stiffer but, at the same time, reduces its strength and deformability under tension.

Such behavior is consistent with the segregated architecture established above. Since the conductive filler is concentrated in interfacial regions rather than uniformly dispersed within the polymer bulk, the continuity of direct polymer–polymer contacts is partially reduced, while the response of the material becomes more strongly governed by the properties of the filler-rich boundaries. As a result, the addition of AmG produces an expected trade-off between electrical/EMI-related functionality and mechanical ductility. At the same time, the obtained mechanical characteristics indicate that the P(VDF-TFE)-based AmG-filled ECPs remain processable and can be considered for applications in which moderate mechanical performance is acceptable, together with enhanced electrical conductivity and microwave attenuation.

It should be noted that fracture-surface SEM analysis was not performed in the present work; therefore, interfacial debonding or local filler aggregation cannot be directly confirmed. The observed decrease in tensile strength is interpreted primarily from the established segregated morphology. In such a structure, filler-rich interparticle regions partially replace direct polymer–polymer contacts and can act as weaker mechanical boundaries and stress-concentration zones during tensile deformation. A detailed fracture mechanism analysis will require additional fractographic studies, which are the subject of our separate upcoming work.

## 4. Conclusions

In this work, aminated graphene was demonstrated as a functional filler for electrically conductive polymer composites with a segregated structure. The applied reductive amination route produced a graphene derivative combining high amine group content, restored sp^2^-conjugated regions, electrical conductivity, and a crumpled morphology favorable for interfacial localization on polymer powder particles.

The structural analysis showed that AmG is distributed over extended particle surface regions and, after hot pressing, forms filler-rich interparticle pathways. This segregated architecture provides percolation-driven conductivity at 1 wt.% filler loading, with composite conductivity reaching approximately 1.3–1.4 × 10^−4^ S/cm at 1000 Hz.

The same segregated conductive architecture also provides microwave attenuation at 11 GHz. For the P(VDF-TFE)-AmG composite, the calculated transmission value was −15.1 dB for a theoretical thickness of 30 mm, corresponding to no more than about 3% transmitted power. Conversion of the logarithmic R/T/A parameters to linear fractions indicates that attenuation is mainly associated with energy dissipation within the composite rather than with surface reflection alone.

Mechanical testing revealed the expected trade-off for segregated conductive systems: the addition of 1 wt.% AmG increased stiffness but reduced tensile strength and elongation at break. Overall, the results identify AmG as a promising low-loading filler for conductive and EMI-attenuating polymer composites, particularly when efficient conductive network formation is required without high filler consumption.

## Figures and Tables

**Table 1:** Summary of the samples prepared and characterized in this work.

Sample	Polymer Matrix	Filler	Filler Content	Processing Route
P(VDF-TFE)	P(VDF-TFE)	-	0 wt.%	Hot pressing
P(VDF-TFE)-AmG	P(VDF-TFE)	AmG	1 wt.%	AmG deposition on polymer powder + hot pressing
PVC-AmG	PVC	AmG	1 wt.%	AmG deposition on polymer powder + hot pressing
PVC	PVC	-	0 wt.%	Hot pressing

**Table 2:** Real and imaginary parts of the complex permittivity and calculated microwave performance parameters for the studied AmG-filled ECPs at 11 GHz (theoretical thickness 30 mm).

Sample	Permittivity, Real Part, ε′	Permittivity, Imaginary Part, ε″	Reflection, dB	Transmittance, dB	Absorbance, dB
P(VDF-TFE)-AmG	3.18 +/− 0.05	0.78 +/− 0.03	−11.9 +/− 0.2	−15.1 +/− 0.3	−0.4 +/− 0.1
PVC-AmG	2.36 +/− 0.04	0.22 +/− 0.02	−13.3 +/− 0.2	−6.9 +/− 0.2	−1.3 +/− 0.1
P(VDF-TFE)	2.72 +/− 0.04	0.33 +/− 0.02	−14.2 +/− 0.2	−4.8 +/− 0.2	−2.1 +/− 0.2

**Table 3:** Comparison with selected aminated-graphene-based and structurally organized graphene/polymer EMI-shielding materials.

Material System	Filler/Architecture	Filler Loading	Conductivity	EMI Frequency/Parameter	Key Difference from This Work	Ref.
This work: P(VDF-TFE)-AmG	AmG-rich segregated interparticle network	1 wt.%	(1.3–1.4) × 10^−4^ S/cm at 1 kHz	11 GHz; T = −15.1 dB at theoretical thickness of 30 mm	Low-loading AmG filler in pressed segregated polymer composite	This work
Ag/polyaniline/AmG textile	Hybrid conductive coating on textile	not directly comparable	not directly comparable	EMI attenuation >40 dB across UHF, L, S, C, and X bands	Textile/metal/conductive polymer hybrid, not bulk segregated polymer composite	[10]
AgNW/PBO nanofiber paper	Ag nanowire/PBO conductive paper	50 wt.% AgNWs	3357 S/cm	EMI SE = 84.1 dB	High-metal-content paper with strong thermal management function	[14]
PLA@graphene modules	3D-printed oriented graphene/polymer modules	not directly comparable	not directly comparable	EMI SE = 41.2 dB	Hierarchical 3D-printed module, not AmG-based segregated powder composite	[22]
CNT/PLA segregated composite	Compressed 3D-printed CNT-coated PLA scaffold	5 wt.% CNTs	not specified	EMI SE = 67.0 dB	CNT-based segregated architecture with higher filler loading	[15]

**Table 4:** Tensile properties of unfilled P(VDF-TFE) and the P(VDF-TFE)-based AmG-filled ECP filled with 1 wt.% AmG. Data are presented as mean ± SD, n = 5.

Sample	Modulus of Elasticity, MPa	Tensile Strength, MPa	Deformation at Failure, %
P(VDF-TFE)	613 ± 24	52 ± 6	346 ± 22
P(VDF-TFE)-AmG (67.2)	674 ± 37	27 ± 2	180 ± 14

## Data Availability

The data presented in this study are available on request from the first author.

## References

[1] Santhamoorthy M., Asaithambi P., Perumal I., Elangovan N., Natarajan P., Lin M.-C., Kim S.-C., Kumarasamy K., Phan T.T.V. (2025). A comprehensive review of the functionalized polymer composite membranes in wastewater treatment. J. Environ. Chem. Eng..

[2] Elele E., Shen Y., Tang J., Lei Q., Khusid B., Tkacik G., Carbrello C. (2019). Mechanical properties of polymeric microfiltration membranes. J. Membr. Sci..

[3] Ong C.S., Lay H.T., Tamilselvam N.R., Chew J.W. (2021). Cross-Linked Polycarbonate Microfiltration Membranes with Improved Solvent Resistance. Langmuir.

[4] Faruk O., Ahmed A., Khadem A.H., Jia L., Sun L. (2025). Graphene-functionalized textile composites for wearable Joule heating applications. Adv. Nanocompos..

[5] Wang E., Wu Y., Islam M.Z., Dong Y., Zhu Y., Liu F., Fu Y., Xu Z., Hu N. (2019). A novel reduced graphene oxide/epoxy sandwich structure composite film with thermo-, electro- and light-responsive shape memory effect. Mater. Lett..

[6] Hasan A.M.M., Susan M.A.B.H. (2025). PEDOT: PSS polymer functionalized carbon nanotubes integrated with graphene oxide and titanium dioxide counter electrode for dye-sensitized solar cells. Heliyon.

[7] Li C., Gong P., Chao M., Li J., Yang L., Huang Y., Wang D., Liu J., Liu Z. (2023). A biomimetic lubricating nanosystem with responsive drug release for osteoarthritis synergistic therapy. Adv. Healthc. Mater..

[8] Reghunath R., Devi K., Singh K.K. (2021). Recent advances in graphene based electrochemical glucose sensor. Nano-Struct. Nano-Objects.

[9] Tajik S., Beitollahi H., Garkani Nejad F., Dourandish Z., Khalilzadeh M.A., Jang H.W., Venditti R.A., Varma R.S., Shokouhimehr M. (2021). Recent developments in polymer nanocomposite-based electrochemical sensors for detecting environmental pollutants. Ind. Eng. Chem. Res..

[10] Akram S., Aziz H., Imran A., Javid A., Nosheen A., Ashraf M., Xue Z., Raza M. (2023). Fabrication of silver/polyaniline/aminated graphene oxide coated textiles for electromagnetic interference shielding application within the different bands of frequency. Synth. Met..

[11] Ren B., Deng Y., Jia Y., Wu X., Feng G., Wang Q., Li H. (2023). Electromagnetic wave absorbing ceramics composites made of polymer-derived SiC with BN@CNTs pyrolyzed higher than 1200 °C. J. Mater. Sci. Technol..

[12] Jiang D., Murugadoss V., Wang Y., Lin J., Ding T., Wang Z., Shao Q., Wang C., Liu H., Lu N. (2019). Electromagnetic Interference Shielding Polymers and Nanocomposites—A Review. Polym. Rev..

[13] Wang Y., Zhao W., Tan L., Li Y., Qin L., Li S. (2023). Review of Polymer-Based Composites for Electromagnetic Shielding Application. Molecules.

[14] Hu Q., Jiang J., Li Q., Zhang H., Liu X., Zhang H., Huang G., Tang L. (2025). Flame-retardant and highly thermally conductive silver nanowires/PBO nanofiber paper for high-performance electromagnetic interference shielding. Mater. Today Chem..

[15] Wang Y., Fan Z.-W., Zhang H., Guo J., Yan D.-X., Wang S., Dai K., Li Z.-M. (2021). 3D-printing of segregated carbon nanotube/polylactic acid composite with enhanced electromagnetic interference shielding and mechanical performance. Mater. Des..

[16] Torkunov M., Shiyanova K., Verkhova E., Yulovskaya V., Ryvkina N., Melnikov V., Gudkov M., Chmutin I. (2023). Powders of rGO-coated polyamide with special electrical properties for SLS 3D printing. J. Compos. Mater..

[17] Shiyanova K., Gudkov M., Torkunov M., Ryvkina N., Chmutin I., Goncharuk G., Gulin A., Bazhenov S., Melnikov V. (2022). Segregated structure copolymer of vinylidene fluoride and tetrafluoroethylene composites filled with rGO, SWCNTs and their mixtures. Polymers.

[18] Shiyanova K., Torkunov M., Gudkov M., Gulin A., Knyazeva A., Ryvkina N., Khashirov A., Rabchinskii M., Chmutin I., Melnikov V. (2025). Surface modification of polyamide by SWCNTs for application in SLS 3D printing. Compos. Part A Appl. Sci. Manuf..

[19] Shiyanova K.A., Gudkov M.V., Gorenberg A.Y., Rabchinskii M.K., Smirnov D.A., Shapetina M.A., Gurinovich T.D., Goncharuk G.P., Kirilenko D.A., Bazhenov S.L. (2020). Segregated Network Polymer Composites with High Electrical Conductivity and Well Mechanical Properties based on PVC, P(VDF-TFE), UHMWPE, and rGO. ACS Omega.

[20] Bheema R.K., Gopu J., Bhaskaran K., Verma A., Chavali M., Etika K.C. (2024). A review on recent progress in polymer composites for effective electromagnetic interference shielding properties–structures, process, and sustainability approaches. Nanoscale Adv..

[21] Chen J., Liu Y.-L., Sun D.-X., Qi X.-D., Yang J.-H., Wang Y. (2024). Recent progress in structural design of graphene/polymer porous composites toward electromagnetic interference shielding application. Chem. Eng. J..

[22] Shi S., Deng S., Jiang Y., Chen J., Sporrer L., Cheng F., Guo Q., Jing J., Chen Y. (2026). Hierarchical Manufacturing of Anisotropic and High-Efficiency Electromagnetic Interference Shielding Modules for Smart Electronics. Nano-Micro Lett..

[23] Suneesh A.S., Vignesh M.V., Ramanathan N. (2026). Functionalized graphene oxide-polystyrene composite microspheres synthesized through in situ polymerization route for efficient uranium removal from sea water conditions. Chem. Eng. Sci..

[24] Rabchinskii M.K., Besedina N.A., Brzhezinskaya M., Stolyarova D.Y., Ryzhkov S.A., Saveliev S.D., Antonov G.A., Baidakova M.V., Pavlov S.I., Kirilenko D.A. (2023). Graphene amination towards its grafting by antibodies for biosensing applications. Nanomaterials.

[25] Stepanova M., Solomakha O., Rabchinskii M., Averianov I., Gofman I., Nashchekina Y., Antonov G., Smirnov A., Ber B., Nashchekin A. (2021). Aminated graphene-graft-oligo(glutamic acid)/poly(ε-caprolactone) composites: Preparation, characterization and biological evaluation. Polymers.

[26] Marsden A.J., Brommer P., Mudd J.J., Dyson M.A., Cook R., Asensio M.C., Avila J., Levy A., Sloan J., Quigley D. (2015). Effect of oxygen and nitrogen functionalization on the physical and electronic structure of graphene. Nano Res..

[27] Prasad G., Yoon J.U., Woo I., Bae J.W. (2023). Fabrication of amino and fluorine functionalized graphene-based polymer composites to enhance the electromechanical conversion efficiency of TENGs for energy-harvesting applications. Chem. Eng. J..

[28] Hithesh M.C., Mohana K.N.S., Harsha Y.M., Sreelakshmi M., Madhusudhana A.M., Kumar M.C.S. (2024). Effects of curing temperature and addition of functionalized graphene oxide on corrosion barrier performance of phenol furfural polymer-amino phenolic resin composite. Prog. Org. Coat..

[29] Glukhova O.E., Rabchinskii M.K., Saveliev S.D., Kirilenko D.A., Barkov P.V. (2022). Aminated graphene nanomesh: Theoretical and experimental insights into process of decorating, topology and electron properties. J. Compos. Sci..

[30] Struchkov N.S., Alexandrov E.V., Romashkin A.V., Silakov G.O., Rabchinskii M.K. (2020). Uniform graphene oxide films fabrication via spray-coating for sensing application. Fuller. Nanotub. Carbon Nanostruct..

[31] Molodtsov S.L., Fedoseenko S.I., Vyalikh D.V., Iossifov I.E., Follath R., Gorovikov S.A., Brzhezinskaya M.M., Dedkov Y.S., Püttner R., Schmidt J.-S. (2009). High-resolution Russian–German beamline at BESSY. Appl. Phys. A.

[32] Stöhr J. (1992). NEXAFS Spectroscopy.

[33] Rabchinskii M.K., Saveliev S.D., Ryzhkov S.A., Nepomnyashchaya E.K., Pavlov S.I., Baidakova M.V., Brunkov P.N. (2020). Establishing the applicability of the laser diffraction technique for the graphene oxide platelets lateral size measurements. J. Phys. Conf. Ser..

[34] Jansen R.J.J., van Bekkum H. (1995). XPS of nitrogen-containing functional groups on activated carbon. Carbon.

[35] Schultz B.J., Dennis R.V., Aldinger J.P., Jaye C., Wang X., Fischer D.A., Cartwright A.N., Banerjee S. (2014). X-ray absorption spectroscopy studies of electronic structure recovery and nitrogen local structure upon thermal reduction of graphene oxide in an ammonia environment. RSC Adv..

[36] Aguilar-Bolados H., Vargas-Astudillo D., Yazdani-Pedram M., Acosta-Villavicencio G., Fuentealba P., Contreras-Cid A., Verdejo R., López-Manchado M.A. (2017). Facile and scalable one-step method for amination of graphene using Leuckart reaction. Chem. Mater..

[37] Zhang W., Ma J., Gao D., Zhou Y., Li C., Zha J., Zhang J. (2016). Preparation of amino-functionalized graphene oxide by Hofmann rearrangement and its performances on polyacrylate coating latex. Prog. Org. Coat..

[38] Rabchinskii M.K., Saveliev S.D., Stolyarova D.Y., Brzhezinskaya M., Kirilenko D.A., Baidakova M.V., Ryzhkov S.A., Shnitov V.V., Sysoev V.V., Brunkov P.N. (2021). Modulating nitrogen species via N-doping and post annealing of graphene derivatives: XPS and XAS examination. Carbon.

[39] Potorochin D.V., Chaika A.N., Molodtsova O.V., Aristov V.Y., Marchenko D.E., Smirnov D.A., Makarova A.A., Walls B., Zhussupbekov K., Walshe K. (2022). Surface functionalization of few-layer graphene on β-SiC(001) by neutral red dye. Appl. Surf. Sci..

[40] Ganguly A., Sharma S., Papakonstantinou P., Hamilton J. (2011). Probing the thermal deoxygenation of graphene oxide using high-resolution in situ X-ray-based spectroscopies. J. Phys. Chem. C.

[41] Rabchinskii M.K., Sysoev V.V., Glukhova O.E., Brzhezinskaya M., Stolyarova D.Y., Varezhnikov A.S., Solomatin M.A., Barkov P.V., Kirilenko D.A., Pavlov S.I. (2022). Guiding graphene derivatization for the on-chip multisensor arrays: From the synthesis to the theoretical background. Adv. Mater. Technol..

[42] Lee V., Dennis R.V., Schultz B.J., Jaye C., Fischer D.A., Banerjee S. (2012). Soft X-ray absorption spectroscopy studies of the electronic structure recovery of graphene oxide upon chemical defunctionalization. J. Phys. Chem. C.

[43] Shnitov V.V., Rabchinskii M.K., Brzhezinskaya M., Stolyarova D.Y., Pavlov S.V., Baidakova M.V., Shvidchenko A.V., Kislenko V.A., Kislenko S.A., Brunkov P.N. (2021). Valence band structure engineering in graphene derivatives. Small.

[44] Mak K.F., Ju L., Wang F., Heinz T.F. (2012). Optical spectroscopy of graphene: From the far infrared to the ultraviolet. Solid State Commun..

[45] Luo Z., Lu Y., Somers L.A., Johnson A.T.C. (2009). High yield preparation of macroscopic graphene oxide membranes. J. Am. Chem. Soc..

[46] Ferrari A.C., Basko D.M. (2013). Raman spectroscopy as a versatile tool for studying the properties of graphene. Nat. Nanotechnol..

[47] Cuesta A., Dhamelincourt P., Laureyns J., Martínez-Alonso A., Tascón J.M.D. (1994). Raman microprobe studies on carbon materials. Carbon.

[48] Seshadri T.R. (1942). Raman effect and hydrogen bonds. Part VII. Study of a few typical carboxylic acids. Proc. Indian Acad. Sci..

[49] Endo M., Kim Y.A., Hayashi T., Muramatsu H., Terrones M., Saito R., Villalpando-Paez F., Chou S.G., Dresselhaus M.S. (2006). Nanotube coalescence-inducing mode: A novel vibrational mode in carbon systems. Small.

[50] Rabchinskii M.K., Ryzhkov S.A., Kirilenko D.A., Ulin N.V., Baidakova M.V., Shnitov V.V., Pavlov S.I., Chumakov R.G., Stolyarova D.Y., Besedina N.A. (2020). From graphene oxide towards aminated graphene: Facile synthesis, its structure and electronic properties. Sci. Rep..

[51] Kirilenko D.A., Dideykin A.T., Van Tendeloo G. (2011). Measuring the corrugation amplitude of suspended and supported graphene. Phys. Rev. B.

[52] Brekhovskikh L.M. (2012). Waves in Layered Media.

